# Can we tackle climate change by behavioral hacking of the dopaminergic system?

**DOI:** 10.3389/fnbeh.2022.996955

**Published:** 2022-10-13

**Authors:** Jérôme Munuera, Eric Burguière

**Affiliations:** ^1^Sorbonne Université, Institut du Cerveau–Paris Brain Institute–ICM, Inserm, CNRS, AP-HP, Hôpital de la Pitié Salpêtrière, Paris, France; ^2^Institut Jean Nicod, Département d’Études Cognitives, École Normale Supérieure (ENS), EHESS, CNRS, PSL University, Paris, France

**Keywords:** dopamine, climate change, reward prediction error (RPE), habit (automatism), reinforcement learning

## Abstract

Climate change is an undeniable fact that will certainly affect millions of people in the following decades. Despite this danger threatening our economies, wellbeing and our lives in general, there is a lack of immediate response at both the institutional and individual level. How can it be that the human brain cannot interpret this threat and act against it to avoid the immense negative consequences that may ensue? Here we argue that this paradox could be explained by the fact that some key brain mechanisms are potentially poorly tuned to take action against a threat that would take full effect only in the long-term. We present neuro-behavioral evidence in favor of this proposal and discuss the role of the dopaminergic (DA) system in learning accurate prediction of the value of an outcome, and its consequences regarding the climate issue. We discuss how this system discounts the value of delayed outcomes and, consequently, does not favor action against the climate crisis. Finally, according to this framework, we suggest that this view may be reconsidered and, on the contrary, that the DA reinforcement learning system could be a powerful ally if adapted to short-term incentives which promote climate-friendly behaviors. Additionally, the DA system interacts with multiple brain systems, in particular those related to higher cognitive functions, which can adjust its functions depending on psychological, social, or other complex contextual information. Thus, we propose several generic action plans that could help to hack these neuro-behavioral processes to promote climate-friendly actions.

## Introduction

Climate change is one of the biggest challenges that human societies will face in the decades to come. Indeed, as has been pointed for many years by the reports of the Intergovernmental Panel on Climate Change, the ecological damage created by this crisis will, and has already, severely limited access to essential resources in many ways. For example, nutrition will be severely impacted through lower agricultural production, and reduced access to water due to severe drought or pollution. Increased extreme temperature variations could also impact the human body’s physiology by reaching the limits of its thermoregulation capacity. Altogether, these events will very likely provoke large migrations of populations trying to escape these threats to find access to basic resources. As a result of mass migrations, conflicts between nations become more likely as available resources become more limited. All these threats are closely linked to survival instincts for which five basic needs have been well-identified. Four of them guarantee the protection of the genes’ survival (Dawkins, 1976): maintenance of energy and nutritional supply (food), fluid balance (water), thermoregulation, and defense of physical integrity (avoiding threat). The purpose of the last one, reproduction, is to perpetuate the survival of the organism’s genes in future generations (Hull, 1943; LeDoux, 2012). Fulfilling these basic needs enables the maintenance of a functional internal physiological condition, enabled by homeostasic regulation (Cannon, 1929, 1939). Indeed, since homeostasis is almost constantly unbalanced (e.g., necessity of regular food intake to compensate calory loss), the organism’s behavior is driven toward the reestablishment of these basic needs to maintain their wellbeing. Therefore, these mechanisms are preserved through evolution and humans, like other animals, will modulate their actions whether these basic needs are fulfilled or not (e.g., fear or happiness) (Ekman and Friesen, 1971; Darwin, 1998; Ekman, 1999; Barrett, 2006). Thus, whether individually or collectively, humans ought to be able to encompass the tremendous threats of climate change and to adjust their behavior to fulfill those needs when they are at stake. Instead, we observe that greenhouse-gas emissions (e.g., fossil fuel and methane emissions), the major source of climate change, continue to rise year after year because of human activity. Our tendency to act on a short-term basis while ignoring disastrous long-term consequences of our behaviors define the climate change paradox. Importantly, here the expression “short-term” is relative to the temporal scale of the climate change (several decades) and can refer to behaviors taking place across days or even months.

In this neuro-educational review we focus on some key behavioral and neural mechanisms, namely the behavioral reinforcement learning process and its underlying dopaminergic system, which could explain this paradox to some extent. We will first summarize and describe the principles of these neurobiological mechanisms, which have been extensively studied in the field of neuroscience for many decades. In a second step, we will suggest various guidelines to help implement corrective policies in order to hack these neuro-behavioral mechanisms and act against the climate change paradox.

## The concept of reinforcement learning and its underlying dopaminergic system

A fundamental question here is to understand why the brain is unable to efficiently learn and generate behaviors that are not detrimental to the climate and by consequence for the agent. In recent years, reinforcement learning algorithms have provided an elegant framework for understanding both how animals optimize their actions to maximize reward and minimize punishment, and how the brain may represent the basic parameters involved in this process (Schultz, 2006; Dayan, 2009). Thus, we will first describe how temporal-difference reinforcement learning model (TDRL) can be implemented in different brain structures such as the basal ganglia (Mirenowicz and Schultz, 1994; Cohen et al., 2012; Lau et al., 2017). Interestingly, we will describe in a second step a neural system which has been identified as key component of reinforcement learning: the dopaminergic (DA) system, also refers to as the “wanting” system. Indeed, when facing positive or negative outcomes, its neuronal discharge mimics what can be expected by a TDRL model (Schultz et al., 1997; Schultz, 2000; Glimcher, 2011; Lau et al., 2017).

## Behavioral mechanisms: Reinforcement learning as a key tool to promote desired actions

In reinforcement learning, feedback is provided to an agent after an action and indicates whether behavior is correct (rewarded) or incorrect (not rewarded or punished). More specifically Sutton and Barto have developed a TDRL (Sutton and Barto, 1998) which postulated that the goal of an agent is to predict the value of future events. The predictions are based on previous experiences where the agent learn to associate temporally a stimulus (e.g., a visual stimulus such as the front door of a restaurant) followed by an outcome such as a food reward. The more reliable this temporal association, the stronger will be the prediction that the (now) conditioned stimulus will deliver an outcome (e.g., the front door of the restaurant predicts that food reward will come shortly). Alternatively, if a reward is missing, this prediction will be weaker next time the conditioned stimulus will be presented.

Thus, TDRL can be used by an agent to adapt behaviors and regulate homeostatic balance by an optimization of reward intake and punishment avoidance. Indeed, because TDRL algorithms learn to predict values associated with perceived states of the environment, they are powerful mechanisms promoting homeostatic regulation by driving the incentives of animals (including humans) toward specific goals of survival and reproductive success. Importantly, these goals can be positive or negative. When they are positive (also refers to as appetitive) such as an economical or food reward, agents will modify their behavior to reach the goal while, when they are negative, they will try to avoid it. In this framework, why would humans not change their behaviors considering the great danger represented by climate change? According to the TDLR model, one could assume that the temporal difference in the context of our action (e.g., taking an airplane) and its consequence (e.g., a rise in global temperature over decades) maybe too long and inconsistent. In this scenario, learning the negative consequences of our actions is not efficient and our behaviors would not be modified. Moreover, neural mechanisms that have been proposed to underlie TDRL, such as the dopaminergic system, are sensitive to time. Thus, the temporal processes engaged in the context of climate change may not be tuned to such a mechanism.

## Promoting appetitive reward seeking and aversive event avoidance: The key functions of dopamine

**FIGURE 1:**
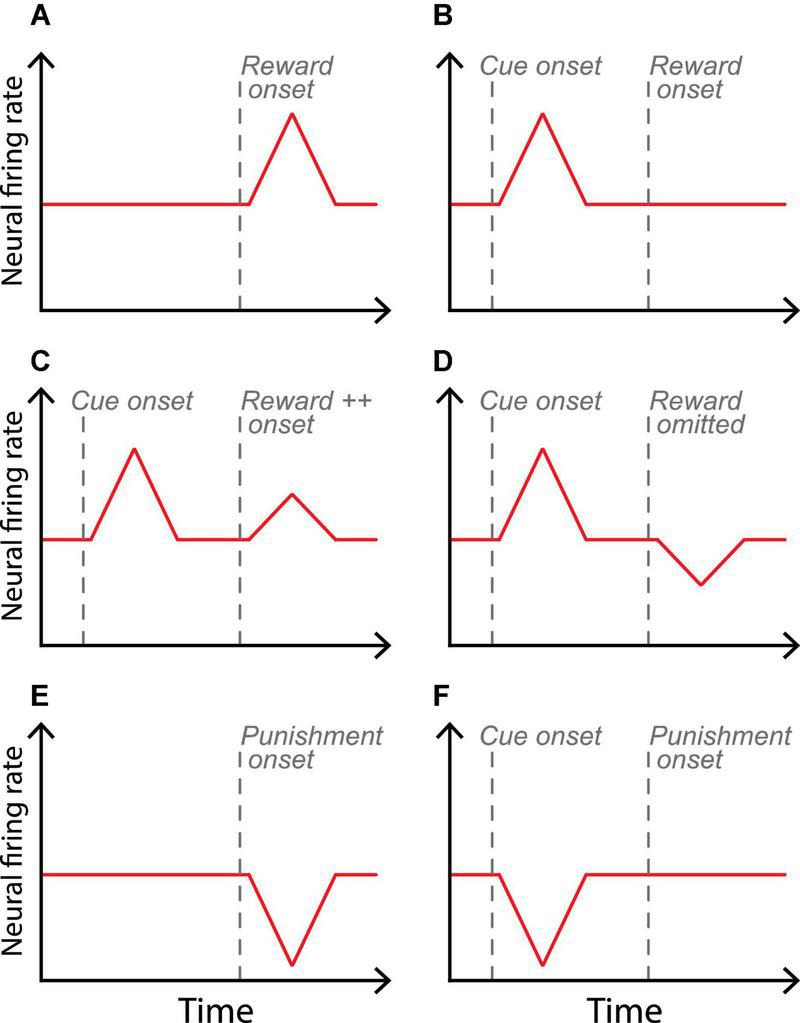
Schematic midbrain dopaminergic neural responses of the encoding of reward prediction errors. Panels **(A,B)** represent neural activity in appetitive contexts where the reward delivery is unexpected **(A)** or predicted by a cue and thus expected **(B)**. Panels **(C,D)** represent firing rates when reward delivery is, respectively, above and below reward expectation. Panels **(E,F)** represent the firing rates of a neuron in aversive contexts where the punishment delivery is unexpected and expected, respectively.

The DA system is composed of neurons located in the midbrain which primarily send massive projections to the prefrontal cortex and the striatum (Haber and Knutson, 2010; Sesack and Grace, 2010) but also to other structures such as the limbic area and the amygdala (Morel et al., 2022). This DA system has been extensively studied and has been proposed to play a central role in encoding expectation of reward delivery (Schultz, 2000). These neurons encode a large range of rewarding experiences (Wise, 2004; Lau et al., 2017) and generate “reward prediction errors,” which consist of the differences between received and predicted rewards. Thus DA neurons can either signal unpredicted outcomes or changes from an expected value (Mirenowicz and Schultz, 1994; Schultz et al., 1997). For example, DA neurons fire a burst of spikes upon reception of an unexpected reward (e.g., fluid, food or money, Figure 1A) or when reward delivery is higher than expected (positive difference, Figure 1C after reward onset). Additionally, when a stimulus has been consistently associated with a reward, DA neurons will be active mainly at the time of the stimulus presentation as it predicts an expected reward (Figure 1B after cue onset). Inversely, when an expected reward delivery is omitted, DA neurons are inhibited in the time interval where reward was expected (negative difference, Figure 1D; Montague et al., 1996). Therefore, as in TDRL algorithm, DA neural activity will be updated at each presentation of the conditioned stimulus, with either a weaker or stronger signal, according to the previous difference between received and predicted rewards. Importantly, several causal manipulations also highlight the direct involvement of DA system in reward learning and motivational processes. For example optogenetics activation or inhibition of DA midbrain neurons during a task where subjects need to learn the value of different stimuli will, respectively increase or decrease the value of the stimuli and ultimately modulate the subjects’ motivation to acquire these stimuli and their associated reward (Stauffer et al., 2016; Van Zessen et al., 2021).

Finally, the opposite neuro-behavioral mechanism can be observed during the presentation of a conditioned stimulus predicting an aversive event. Indeed, DA neurons receive projections from other sub-cortical area, such as the lateral habenula. This area encodes primarily aversive events in a similar manner to what is observed in DA midbrain neurons for appetitive events (e.g., lateral habenula neurons increase their firing rate upon reception of an unexpected punishment) (Baker et al., 2016). Thus, thanks to the circuitry involving mutual connections between the midbrain DA neurons and the lateral habenula, DA neurons will be inhibited by the lateral habenula upon reception of an unexpected negative outcome (Figure 1E) or upon presentation of conditioned stimuli predicting a punishment (Figure 1F; see Schultz, 2016 for a review). Thus, while waiting for the unavoidable punishment, the subject will typically enter in a state of behavioral freezing associated to anxiety (Kumar et al., 2013). However, this state of fear can be reduced when the organism performs an adapted action to avoid the threat of a punishment. This reduction of fear could act as reinforcer for the DA system which will encode it as a positive stimulus (i.e., similarly to Figure 1B) since avoiding a negative outcome is something positive for an organism. Therefore, in line with TDRL, the avoiding action will be promoted when the predictive punishment stimulus is re-experienced (Gentry et al., 2016, 2019; Lloyd and Dayan, 2016).

The DA system should be particularly well-adapted to promote beneficial behavior and avoid detrimental action for the agent. Such a biological mechanism would be appropriate in the context of climate change since we are more and more aware of the ecological costs and benefits of our individual decisions; with our action leading to either negative (e.g., take a plane, buy a house, etc.) or positive (e.g., insulate the house, use public transport) consequences in term of greenhouse-gas emissions. Unfortunately, DA system and reward processing are influenced by another factor, time discounting, which could account for the lack of human motivation to protect climate.

## Outcome values and dopamine responses are sensitive to time

When investigating outcome value, amplitude and probability of reward are traditionally used. However, the time between a predictive conditioned stimulus and reward delivery has been historically identified as a major factor modulating the subjective value of reward (Critchfield and Kollins, 2001; Frederick et al., 2002; Hayden, 2015). Using a classical task design involving choice between a small but rapidly available and a large but delayed reward, experimental psychologists and neuro-economists have more recently formalized that birds (Ainslie, 1974; Rodriguez and Logue, 1988), rodents (Richards et al., 1997), monkeys (Kobayashi and Schultz, 2008), and humans (Rodriguez and Logue, 1988) generally favor small and rapidly available reward. This suggests that the value of reward decreases as a function of time, a process known as the time discounting effect (also refers to as temporal or delay discounting). Therefore, this process will favor sub-optimal short-term rewards rather than more rewarding options available on a longer timescale. The time discounting effect applies primarily to positive outcomes but can also bias negative outcomes, the latter being perceived as less aversive in the future than in the present (Stevenson, 1986; Critchfield and Kollins, 2001; Frederick et al., 2002; Estle et al., 2006; Madden and Bickel, 2010).

**FIGURE 2:**
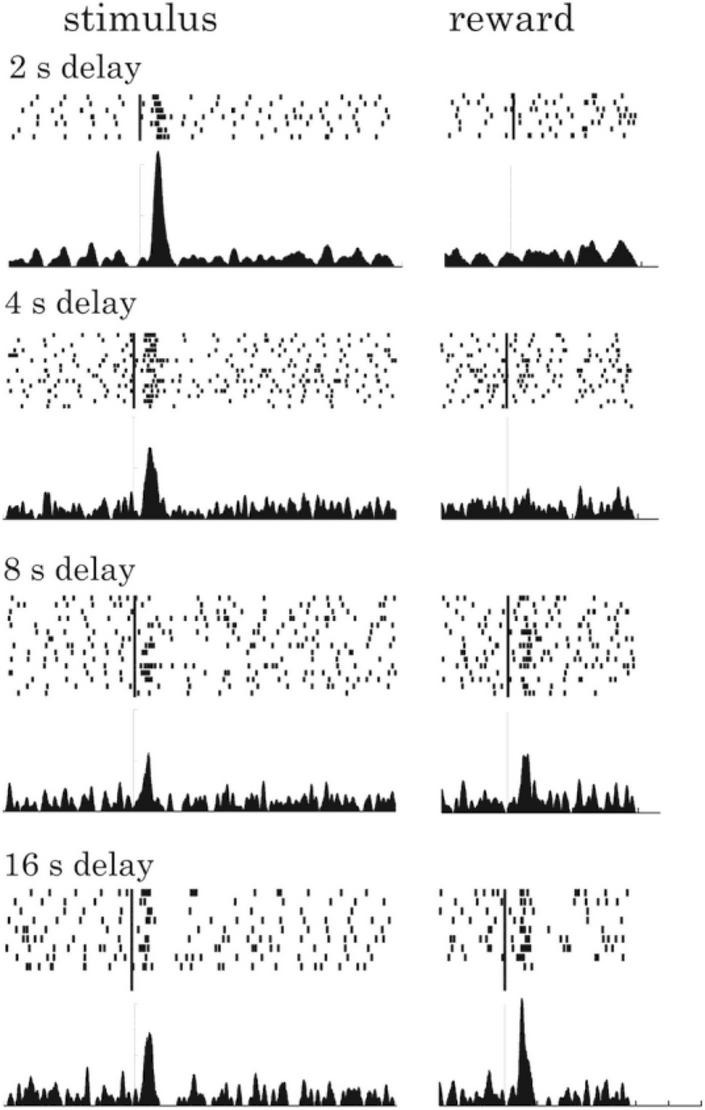
Time discounting and dopamine discharge of a single dopaminergic neuron at conditioned stimulus **(left panels)** and reward **(right panels)** onsets for each delay condition. These delays range from 2 s **(upper panel)** up to 16 s **(bottom panel)** between conditioned stimulus and reward delivery (note that reward quantities were equated between stimuli). Peri-stimulus time histograms represent the average neuronal discharge in each condition. On top of each panel, black tick marks of raster plots identified times of neuronal impulses. Neural responses decrease as a function of time between the conditioned stimulus onset and the reward delivery. Interestingly, dopaminergic responses at time of the reward delivery increased with longer delays, possibly reflecting an unexpected reward occurrence as if the temporal link between the predictive stimulus and reward was absent. Adapted from Kobayashi and Schultz (2008). Copyright 2008 Society for Neuroscience.

Time discounting has been established behaviorally thanks to subject choice, but, there is strong evidence that also shows that the DA system does indeed discount reward as a function of time. In a landmark paper, Kobayashi and Schultz (2008), recorded DA midbrain neural activity using a simple time discounting task design. Non-human primates were trained to associate several visual stimuli, each predicting reward delivery (juice) at different time intervals. At the time of conditioned stimulus onset, reward delay associated with the different stimuli decreased the DA neurons responses (predicting the future reward outcome) according to a decay function similar to that observed in choice behavior (Figure 2).

It might also be the case that short-term outcomes are more valuable because the nervous system directs behavior toward the current, most unbalanced basic needs, in order to regulate homeostasis, thus minimizing uncomfortable physiological and psychological states. Altogether, the time scale into which humans perceived that their behavior would affect climate, and thus their wellbeing, is way too long and therefore the DA system may not be well-adapted to estimate the negative consequences of our actions. On the contrary, this system would favor impulsive behaviors toward short-term appetitive outcomes, which are often detrimental to the climate.

## Involvement of the dopaminergic system in the development of habitual behaviors

Dopaminergic neurons encode several parameters related to the stimulus value but not necessarily related their exact nature. The output structures of the midbrain DA neurons are therefore important if we wish to understand how different reward and punishment encoding could be processed by the brain. A key output structure of DA neurons within the basal ganglia is the striatum. Like other basal ganglia areas, this structure can be divided in three main territories, all receiving massive DA projections. First there is the limbic territory in the ventral section of the striatum which receives its DA inputs primarily from the ventral tegmental area (VTA). The associative and sensorimotor territories are localized, respectively in caudate and putamen, which constitute the dorsal striatum. Unlike the limbic system, these territories receive DA inputs primarily from the substantia nigra pars compacta (SNc), the second main area together with the VTA containing DA neurons in the midbrain. These three territories are involved in goal-directed behavior where the limbic system provides the organism’s motivation by setting the goal of the action, while the associative system is involved in action selection to reach the correct goal. Finally the sensorimotor system defines the details of the action execution by selecting the correct movement (s) leading to the achievement of the goal-directed action (for a review, see Tremblay et al., 2015). These circuits are recruited to different extents over time: while the ventral and dorsomedial striatum are important, especially during the initial acquisition of a goal-directed behavior, it is the dorsolateral striatum that is crucially involved when the behavior becomes automatized and habitual (Alexander et al., 1986; Yin and Knowlton, 2006; Graybiel, 2008). Interestingly, DA signaling shows the same progressive recruitment of ventral, dorsomedial and then dorsolateral striatum over the time course of habit formation. Indeed, phasic DA release was initially observed in the ventromedial striatum on presentation of the reward-predicting conditioning stimulus, but over time, when the behavior becomes habitual, DA release progressively emerges in the dorsal striatum while declining in the ventral part (Willuhn et al., 2012; van Elzelingen et al., 2022). Thus, DA system seems to play a crucial role in promoting habit formation over time when an action is promoted by the repetition of its positive or negative feedback elements. Habitual behaviors are very efficient at automatizing actions leading to the fulfillment of our basic needs, which, while non-problematic when punctual (e.g., eating occasionally junk food) could be detrimental for climate change when these actions become daily habits (e.g., automatically turning on light/heating when arriving home, binge eating junk food while watching TV, etc.). Since habits are characterized by a lack of flexibility and need a strong inhibition to prevent their expression, they could be seen as another obstacle to the fight against climate change.

## Using the current neuroscientific knowledge to suggest action that might help mitigate climate change

We have shown in the first section that time discounting effects can favor decisions to acquire a relatively small but immediately available outcomes (e.g., eating an appetitive tasty beefburger or using a plane to maximize travel time) instead of a large but delayed outcomes (e.g., preventing climate change to promote survival and wellbeing in the long-term). Therefore, the DA system and its associated brain areas have been certainly very useful throughout evolution to guarantee our short-term survival by adapting behaviors to rapidly fulfill our basic needs. At first sight, they may seem not to be adapted to the fight against climate change where negative consequences will be observed only after decades.

However, some solutions and complementary neural mechanisms should be considered to hack, by-pass, or regulate this short-term based biological system. Indeed, according to TDRL, being able to perceive any positive consequence of climate-friendly action (even if the main goal of the action is not to protect climate) should trigger the DA system to learn and strengthen the association between the action and its positive consequences. This subsequent reinforcement will then promote the repetition of the same type of behavior. Therefore, all climate-friendly behaviors that can be reinforced by any means should be encouraged by policy makers. Based and inspired by these observations, we are proposing few suggestions in this second section that could help adopt climate-friendly behaviors and to avoid climate-detrimental actions.

## Suggestion 1: Promote climate-friendly behavior with short-term positive (environmental) feedback

A first option to promote climate-friendly action is to provide short-term positive feedback associated with the agent action. This sounds like a complex process since the behavioral consequences of climate-friendly behavior can, most of the time, only be experienced on a long-term basis. However, one could hack this limitation by finding more immediate incentives as positive outcomes. For example, many of these climate-friendly actions can be positively promoted on an ecological perspective by promoting biodiversity. Indeed, this dimension can be used to reinforce subject behaviors since biodiversity improvement can potentially be experienced on a much shorter time scale, i.e., change in biodiversity can be experienced after few months, sometimes even weeks instead of several decades. Because temporal discounting can happen across several years in human with remaining (although very small) value, the shorter time range of biodiversity change should be enough to provide a salient positive feedback related to the subjects’ virtuous behaviors (Chapman, 1996). Moreover, while climate change is a major driver of biodiversity degradation, biodiversity loss can also increases climate change thus creating a vicious circle (e.g. Corlett, 2020). Therefore while serving the same purpose of curving greenhouse-gas emission, promoting biodiversity friendly action by individuals (e.g., revegetation or city gardening which increase re-uptake of carbon dioxide and temper heat waves thus limiting the use of air conditioning), can be encoded by the DA system in a more salient way since it is experienced on a shorter time range.

Moreover, improving biodiversity, whether at the individual or institutional level, could be used to promote climate-friendly behaviors. As an example, the creation and maintenance of nature enriched cycle lanes or public transportation can be experienced immediately by citizens and give positive feedback while traveling by bike or train. Many options are probably available to implement such kind of strategy, but the take home message is that feedback after an attempt to improve biodiversity should be experienced by the agents as soon as possible. This will maximize the positive feedback detection by the DA system and reinforce the associated climate-friendly behavior. Indeed, improving biodiversity by creating a healthier ecosystem could also improve humans’ wellbeing, a concept sometime refers to as biophilia which hypothesizes that humans have an (innate) affinity for living organisms and systems (Wilson, 1986). While the innate nature of biophilia is debated (Joye and de Block, 2011) there is nonetheless much evidence that rich ecosystems decrease anxiety and improve wellbeing which highlight its appetitive reinforcing dimension, thus making biodiversity conservation a good tool to promote climate-friendly action (Sandifer et al., 2015). Finally protecting the ecological environment could also enhance the health of residents (e.g., thanks to the improvement of air and water quality), another positive outcome that can also be experienced in the shorter-term.

Using the same concept where an individual’s behavior could be modified to more climate-friendly actions, even implicitly, is the non-coercive intervention on choice architectures, commonly referred to as “nudges.” They can be used in the context of climate change by trying to modify citizen behavior toward climate-friendly actions (Siipi and Koi, 2022). The general idea is to “trick” people on using alternative climate-friendly means, such as climbing stairs rather than using the elevator, by providing incentive feedbacks (e.g., write cumulative calories loss on the final step of the stairs). Nudges are easy to implement, are low-cost and promote choice alternatives to various personal and societal issues, without banning other options, which can lead to their widespread adoption.

## Suggestion 2: Promote climate-friendly behavior through intrinsic instead of extrinsic motivation

As mentioned above, the DA system and outcome value encoding are usually investigated using basic extrinsic rewards such as food, liquid or money, i.e., those that are available in the environment. However, evidence suggests that another type of reward, can motivate subjects to perform an action by achieving an intrinsic need. Intrinsic rewards (Ryan and Deci, 2000c), do not necessarily lead to achievement of extrinsic goals but trigger intrinsic (or autonomous) motivation (Nix et al., 1999; Ryan and Deci, 2000a) and promote wellbeing. They are thus different from extrinsic rewards, which are closely related to the basic needs, available in the environment and promote controlled (or extrinsic) motivation. Interestingly, controlled motivation can trigger anxiety in the subject performing an action, not because the subject *wants* to do it but *must* do it. This concept is linked to a popular framework developed by Ryan and Deci known as self-determination theory (Ryan and Deci, 2000b). This theory postulates that agents can gather inherent satisfaction derived from action. These authors have initially identified two needs: autonomy and competence and suggest that these are intrinsically motivational because they improve control over the environment (Ryan et al., 2006). Thus, being able to choose and control one’s own action could be a form of intrinsic reward. Indeed, several animal species, including humans, consistently prefer contexts with opportunities to choose compared to those without, even when making a choice affords no improvement in the final outcome (Bown et al., 2003; Egan et al., 2007; Leotti et al., 2010). For example, in one experiment, subjects were asked to purchase one of their preferred items in a two-stage decision task. They were first asked to choose between two options, one with no choice that provides a relatively inexpensive preferred item, and another with the opportunity to choose between a non-preferred item and a preferred item but which is more expensive than in the first option. Surprisingly, subjects tend to choose the second option, at extra cost, precisely because they were able to choose it (Bown et al., 2003). It has been recently suggested that the DA system may be involved in processing such intrinsic rewards (Schultz, 2000; Bromberg-Martin and Hikosaka, 2009). This idea is supported by previous studies, as for example in a study where humans expressing more DARPP-32, a gene linked to DA plasticity, show a stronger bias for choosing items they were free to explore compared to items they were forced to sample (Cockburn et al., 2014). It has also been shown that another type of intrinsic reward, information-seeking, is encoded by the DA system. Indeed, DA neural activity of primates performing a conditioning task has been shown to be stronger during the presentation of a cue predicting information compared to a cue predicting the absence of information (Bromberg-Martin and Hikosaka, 2009). Taken together, these behavioral and neurophysiological data suggest that, when implementing a behavioral policy promoting climate-friendly actions, it is important to implicate citizens in such a process in order to make them feel in control of their own behavior. Otherwise, restrictive or punitive policies would trigger negative responses from people who would decide to avoid climate-friendly behavior in order to regain control. In other words, promotion of climate-friendly behavior should rely on situations where the subjects feel that they make choices rather than feeling under obligation to perform them.

Finally a third requirement of self-determination theory, referred to as “relatedness,” has been postulated by the authors, which is the feeling of being connected with others (La Guardia et al., 2000; Ryan and Deci, 2001). Here, social interactions are also rewarding. Thus it has been suggested that climate-friendly behavior promoted by one’s peers can encourage other to shift toward the same type of behavior (Boon-Falleur et al., 2022). Since the DA system has also been identified in social behavior encoding (Klein and Platt, 2013; Gunaydin et al., 2014; Munuera et al., 2018), using social strategies to curve climate change sounds like a valid approach. However, these strategies probably have to be used with caution to avoid the risk of the negative signaling of value judgment—a term associated with someone publicly displaying their actions only to improve their social reputation (Geoffrey, 2019).

## Suggestion 3: Provide a short-term positive economic value for climate-friendly behavior

Based on the known DA system properties, the third suggestion relies on neuro-economical strategies. These types of strategies are already implemented by many actors, but it is important to emphasize that they are indeed adapted on a neuro-behavioral perspective and should therefore be promoted to generate goal-directed behavior toward items with a minimal impact on climate. In general, neuro-economy suggests that increasing the relative extrinsic value of an item with low negative impact on climate relies either on decreasing its cost or on increasing its benefit. The options here are endless but depend on institutional willingness to implement economic policies toward climate-friendly actions in others to trigger the DA system to associate these actions as something economically more valuable.

For example, it should be trivial, but very powerful for the DA system, to decrease the cost of goods produced with lower greenhouses gas emitting methods by reducing their tax rates. Another alternative is by adding bonuses, defined here as an additional reward, for choosing options which minimize greenhouse-gas emissions (e.g., accumulation of “miles” while traveling by train). Thus, subjects’ DA systems will promote the association of these items to an extra value given by the benefit of the bonus which will subsequently promote this type of behavior. Another advantage of bonuses is that the subject will not feel *compelled* to do anything in particular since other options will still be available (e.g., travel by plane, car, etc.). Subjects will thus be more intrinsically motivated to select climate-friendly actions (promoted by bonuses) since they will have the option to choose it.

## Suggestion 4: Promote educational and cognitive tools to regulate climate-detrimental behaviors

Up to this point we have specifically described the involvement of the DA system in learning and promoting actions toward short-term reward. However, the human species has developed a large prefrontal cortex. Among its many functions, the prefrontal cortex is involved in behavioral inhibition and in the regulation of impulsive behaviors (Aron et al., 2004; Winstanley et al., 2004; Chamberlain and Sahakian, 2007; Eagle et al., 2008; Madden and Bickel, 2010) such as avoiding the choice of rewards that are “pleasant” in the short-term but detrimental in the long-term (e.g., avoiding eating junk food to improve general health). The prefrontal cortex generally acts by imposing a cortical drive on sub-cortical structures to actively inhibit and regulate default strategies, in order to optimize decision-making and to find better alternatives. Therefore, we are potentially adapted to mitigate our actions so long as we understand that they will surely and negatively impact our lives or the ones of future generations in the case of climate change. To maximize this process, it is crucial to introduce or strengthen climate education especially with children. Education provides an essential intrinsic motivational framework that will participate in the processes during decision-making in our daily lives. For the topic of climate change, several educational strategies can be implemented: For example long-term environmental consequences of climate change; concepts of sustainable environment; negative social impact of climate degradation, and many others. Providing these educational cognitive tools early in life can make a decisive difference in the developing brain that will shape individual’s mind. In particular, promoting climate-friendly actions intentionally chosen early in life would become, by repetition, habitual in the long-term. All along this process, the DA system which remains, as described extensively above, a reward system in the learning process will help to create good habits for both the individual, society and the climate.

Thus, these educational policies will not only provide cognitive tools to understand and fight the climate crisis but also prevent ingraining bad habits detrimental for the climate, which are then difficult to modify once in place.

This last suggestion could be widely developed since the neuroscientific literature on cognitive inhibitory control is extensive and well-documented. Importantly, it proposes that if institutions act substantially and rapidly to provide educational tools, either at school or even accessible to a broader public, the implementation of such climate-friendly policies could be particularly efficient, perhaps on a longer term but with lasting effects.

## Conclusion

Only few years remain for us to change our behavior, and more generally our model of society, in order to avoid the wide ranging, disastrous consequences, of climate change. Due to time discounting effect and the requirement to survive on a relative short-term basis (i.e., vs. the climate temporal scale), humans can mistakenly be defined as climate skeptics and in denial of the future detrimental consequences of their short-term actions. From a neurobiological perspective, this skepticism may rely to some extent on neurophysiological mechanisms such as the DA system, which gives a stronger value to outcomes delivered at a short-term interval. Understanding the generic function of such neural mechanism may help us reconsider their utility to generate behavioral self-adjustments in favor of climate-friendly actions. Moreover, the brain is a complex machine and other neural mechanisms come into play when addressing the question of climate change mitigation. Thus, acknowledging and raising awareness of the underlying brain processes controlling our climate-detrimental behavior may help citizens and policy makers to hack those systems and implement adapted solutions. It will be difficult but *it is possible* as proven by behaviors that are already changing at the individual and collective levels, although not fast enough. Importantly, biological mechanism that apply at the individual level are not necessarily useful for our understanding of the complex dynamics of the different groups constituting human societies. Thus, additional complementary expertise, spanning from cognitive neuroscience to social and economic sciences, are also necessary to comprehensively address the problem of climate change. There is no easy solution, but we believe that understanding the neural correlates decreasing human willingness to efficiently mitigate climate change is crucial to implement the necessary behavioral modification at both the individual and collective levels. There is in fact no strong evidence of a neuro-behavioral curse of climate change and we all potentially have the solution to counter it.

## Author contributions

JM and EB contributed to the original idea and the plan of the review manuscript. JM wrote the first draft of the manuscript. EB edited the manuscript and wrote sections of the manuscript. Both authors contributed to the manuscript revision, read, and approved the submitted version.
